# Preparation and Properties of Elastic Mullite Fibrous Porous Materials with Excellent High-Temperature Resistance and Thermal Stability

**DOI:** 10.3390/ma17133235

**Published:** 2024-07-01

**Authors:** Xiang Zhang, Xueying Zhang, Zhongyan Wang, Yunjia Xue, Anran Guo, Liwen Yan, Feng Hou, Jiachen Liu

**Affiliations:** 1 Key Lab of Advanced Ceramics and Machining Technology of Ministry of Education, School of Materials Science and Engineering, Tianjin University, Tianjin 300072, China; zx_koj@163.com (X.Z.); 18131126553@163.com (Z.W.); arguo@tju.edu.cn (A.G.); lwyan@tju.edu.cn (L.Y.); houf@tju.edu.cn (F.H.); 2 Beijing Institute of Astronautical System Engineering, Beijing 100076, China; 3 Beijing Hangxing Machinery Co., Ltd., Beijing 100013, China; xueyj@tju.edu.cn

**Keywords:** ceramic fiber, thermal stability, fibrous porous ceramic, thermal insulation performance

## Abstract

Mullite fiber felt is a promising material that may fulfill the demands of advanced flexible external thermal insulation blankets. However, research on the fabrication and performance of mullite fiber felt with high-temperature resistance and thermal stability is still lacking. In this work, mullite fibers were selected as raw materials for the fabrication of mullite fibrous porous materials with a three-dimensional net structure. Said materials’ high-temperature resistance and thermal stability were investigated by assessing the effects of various heat treatment temperatures (1100 °C, 1300 °C, and 1500 °C) on the phase composition, microstructure, and performance of their products. When the heat treatment temperature was below 1300 °C, both the phase compositions and microstructures of products exhibited stability. The compressive rebound rate of the product before and after 1100 °C reached 92.9% and 84.5%, respectively. The backside temperature of the as-prepared products was 361.6 °C when tested at 1500 °C for 4000 s. The as-prepared mullite fibrous porous materials demonstrated excellent high-temperature resistance, thermal stability, thermal insulation performance, and compressive rebound capacity, thereby indicating the great potential of the as-prepared mullite fibrous porous materials in the form of mullite fiber felt within advanced flexible external thermal insulation blankets.

## 1. Introduction

During long-term flight and re-entry processes, advanced aircraft will be severely affected by aerodynamic heating environments [1,2,3]. Lightweight, high-temperature-resistant, and reusable thermal protection systems (TPSs) that are non-ablative, efficient in the long term, highly reliable, and cheap to maintain will fulfill the criteria of aerospace vehicles. In the future, advanced aircraft will travel at higher speeds and with greater mobility. Therefore, enhancing the high-temperature thermal stability and thermal insulation performance of TPSs [4,5] is crucial for further improving the technological capacity of aerospace vehicles [6,7,8].

Flexible external thermal insulation blankets, which are commonly applied in temperatures below 1000 °C, are crucial within large-scale ceramic passive thermal protection materials on the surfaces of aircraft [9,10] and have attracted significant research attention in recent years. In general, they are mainly composed of fiber felt, fiber fabrics, and fiber threads. Fiber felt accounts for over 80% of the volume of flexible external thermal insulation blankets, and it possesses a porous skeleton formed by numerous overlapping fibers [11,12,13]. Due to its unique structure featuring high porosity and low bulk density, fiber felt plays an important role in the high-temperature thermal stability, thermal insulation, and compression rebound performance of flexible external thermal insulation blankets [14,15,16].

The composition of the fiber system will have a significant impact on the performance of a given product [17,18,19]. Flexible reusable surface insulation (FRSI) [20], an early type of flexible external thermal insulation blanket, is formed using aramid fibers and is most commonly used at temperatures below 500 °C. Advanced flexible reusable surface insulation (AFRSI) involves silica fiber felt, which is then wrapped with silica fiber fabrics and sewed with silica fiber threads to fix the entire flexible structure. Benefiting from the excellent high-temperature resistance of silica fibers, aircraft covered with AFRSI can withstand temperatures above 800 °C. This product exhibits extremely low thermal conductivity (~0.033 W·m^−1^·K^−1^) at room temperature and under atmospheric pressure. Due to its excellent thermal insulation performance, AFRSI is commonly used in X-51A hypersonic aircraft (which achieve a flight speed of up to 5 Mach) [21,22]. Based on AFRSI, researchers have successively developed two alternative materials for thermal protection. One is composite flexible blanket insulation (CFBI), which is prepared from silica fiber felt and silicon carbide fiber fabric and features a multi-layer structure formed by reflective film. The room-temperature thermal conductivity of CFBI is about 0.035 W·m^−1^·K^−1^, which is slightly higher than that of AFRSI; that said, its temperature resistance is significantly superior. The other material is tailorable advanced blanket insulation (TABI) made from silicon carbide fiber fabrics [23]. This insulation material is filled with aluminum borosilicate and alumina felt and features a triangular prism structure. CFBI and TABI can both further enhance the high-temperature resistance of advanced aircraft. In the 21st century, conformal reproducible insulation (CRI) [24] was first proposed, to be achieved using ceramic fiber felt composites—including silicon oxide, alumina, and boron oxide fiber felt—in the middle layer. At the same time, aluminum borosilicate fiber fabrics were applied to high-temperature surfaces, and silica fiber fabrics were applied to low-temperature surfaces, serving different purposes and functioning in a variety of industries. In addition, CRI involves the application of a ceramic coating (with high-temperature resistance and erosion resistance) to the outermost layer of an original flexible thermal protection material. Therefore, aircraft covered with CRI can withstand temperatures of up to 1200 °C, and CRI has been successfully applied to the surface of orbital test aircraft X-37A and X-37B. Multi-layered insulation (MLI) with a multi-layer reflective structure is undertaken using zirconia fiber felt and silica aerogel. Aircraft covered with MLI can withstand up to 1600 °C due to the excellent high-temperature resistance of zirconia fiber [25].

Oxide ceramic fibers have excellent high-temperature thermal stability, oxidation resistance, and good physical and chemical properties even after long-term exposure to demanding high-temperature oxygen-rich environments, making them an outstanding raw material for ceramic fiber felt [26,27,28,29]. Although silica fiber felt, showing good thermal insulation performance, is the most commonly applied material in oxide ceramic fiber felts, its operating temperature is relatively low. Alumina fiber felt, aluminum borosilicate fiber felt, and zirconia fiber felt have better high-temperature resistance, but also usually have higher thermal conductivity and incur higher costs. Mullite (3Al_2_O_3_·2SiO_2_), which is the only stable binary compound in the alumina–silica system, possesses excellent high-temperature resistance, thermal shock resistance, and chemical corrosion resistance, whilst also demonstrating low thermal conductivity and high-temperature creep and coming at low cost [30,31,32]. Therefore, it is a promising ceramic fiber felt material that can meet the requirements of advanced flexible external thermal insulation blankets. However, there has been limited research conducted on the fabrication and performance of mullite fiber felt with good high-temperature resistance and thermal stability.

Among diverse methods for fabricating ceramic fiber felt [33,34,35], since wet processes can efficiently prepare complex-shaped ceramic fiber felts and are beneficial when modifying and controlling the structure of ceramic fiber felts during preparation, they are considered an ideal choice in the preparation of ceramic fiber felts [36,37].

In this work, considering the differences in the composition of commercial mullite fibers and theoretical mullite fibers, mullite fibers and alumina–silica fibers (which can be transformed into a mullite phase at high temperatures in the range of 1200–1400 °C) [38,39,40] with low thermal conductivity and good heat resistance and corrosion resistance were selected as the raw materials in this study. Using a combination of the molding method and vacuum filtration method, mullite fibrous porous materials with a three-dimensional porous skeleton were prepared. In addition, the resulting high-temperature resistance and thermal stability were systematically investigated by analyzing the effects of heat treatment at different temperatures on the phase composition, microstructure, and performance of the as-prepared alumina–silica fibrous porous materials.

## 2. Materials and Methods

### 2.1. Materials and Experiment Process

Three kinds of mullite and alumina–silica fibers were purchased from the Shanghai Institute of Ceramics, Chinese Academy of Sciences (Shanghai, China) and denoted ASF1, ASF2, and ASF3, respectively. More details of these alumina–silica fibers are shown in Table 1. The sodium hexametaphosphate (SHMP, Aladdin Industrial Co., Ltd., Shanghai, China) was used as a dispersant to improve the dispersibility of alumina–silica fibers in slurry [41].

**Figure 1:**
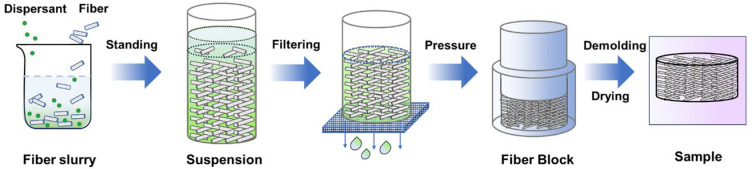
Process of fabricating mullite fibrous porous materials.

The process of preparing mullite fibrous porous materials is shown in Figure 1. Undertaking a typical synthesis process, pretreated ceramic fibers with an aspect ratio of 500–800 were obtained using a high-speed pulper (2890 r/min). Subsequently, said pretreated ceramic fibers (0.2 wt%), deionized water, and SHMP (0.4 wt%) were mixed for 30 min using magnetic stirring to obtain a fiber slurry. Then, the slurry was poured into a cylindrical metal mold (Φ100 mm) and left to stand for 1 h. After the fibers were evenly and stably distributed in the suspended slurry, a significant portion of the solution was subsequently filtered through a mesh screen, resulting in fiber overlapping and the formation of a fiber block. Then, the fiber block was covered with a metal block, which applied a pressure of 0.1 MPa to further facilitate the filtration of the residual solution. After demolding, the fiber block was dried at 65 °C for 12 h to obtain the desired alumina–silica fibrous porous material. The alumina–silica fibrous porous materials prepared using ASF1, ASF2, and ASF3 were denoted ASFPM1, ASFPM2, and ASFPM3, respectively. The products were calcined at 1100 °C, 1300 °C, and 1500 °C for 2 h, and the calcined products were tested to explore their composition, microstructure, and properties after high-temperature treatment.

### 2.2. Characterization

**Figure 2:**
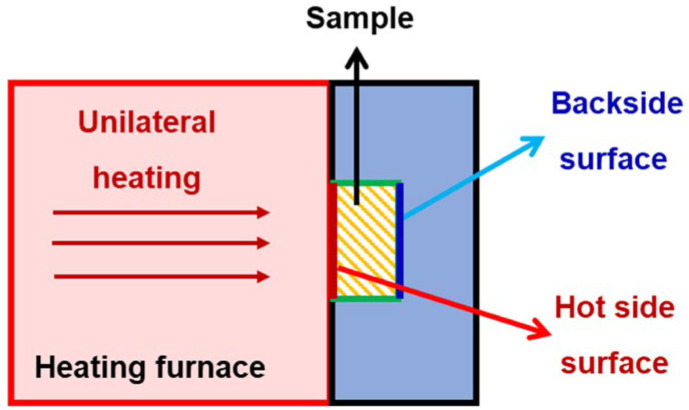
Schematic diagram of backside temperature testing.

The phase compositions of the products with and without heat treatment (1100 °C, 1300 °C, 1500 °C) were analyzed using X-ray diffraction (XRD) (Cu K_α_ radiation, MiniFlex600, Rigaku Co., Tokyo, Japan) over a 2θ range of 5 to 90° at room temperature. Based on the XRD data, the phase quantification of the products was analyzed using Jade 6.0 (Materials Data, Inc., Aubrey, TX, USA). The microstructure of the products was observed via a field emission scanning electron microscope (FESEM) (S4800, Hitachi Ltd., Tokyo, Japan, test voltage of 5 kV~15 kV). The bulk density was calculated by measuring the geometry volume and mass of the samples. The thermal conductivity values of the products were determined using the hotwire method and a thermal conductivity coefficient analyzer (TC3200, Xi’an Xiatech Electronics Co., Ltd., Xi’an, China) at room temperature. Compressive rebound tests were carried out with an electronic universal testing machine (CMT4303, Meister Industrial Systems, Shenzhen, China) with a loading speed of 0.5 mm·min^−1^. Thermal insulation performance was analyzed through a backside temperature test carried out by means of a heating furnace (KSL1700X, Hefei Kejing Co., Ltd., Hefei, China). During the backside temperature test, the heating furnace uses radiation heat as its heat source, with a heating rate of 10 °C·min^−1^. After reaching a set temperature (i.e., the test temperature), the sample is placed in the furnace and heated consistently and unilaterally in the air for 4000 s. The surface of the sample heated by the furnace is termed the hot side surface. Opposite the hot side surface of the sample is the backside surface, which is connected to a backside temperature sensor. This device can simultaneously record the set temperature, the hot side temperature, and the backside temperature in real-time, as shown in Figure 2.

## 3. Results

### 3.1. Dispersibility, Bulk Density, and Thermal Conductivity of Alumina–Silica Fibers

In the process of fabricating alumina–silica fibrous porous materials, the dispersibility of fibers in the slurry is one of the key factors affecting the uniformity of the fibrous porous structure, which in turn affects the various properties of the products [42,43]. However, due to the poor hydrophilicity of fibers, it is usually difficult for fibers to disperse uniformly in aqueous solutions, leading to fiber entanglement and aggregation. Poor dispersibility of fibers will lead to the poor stability of the fiber slurry [44,45], resulting in defects such as a rough surface, uneven structure, and poor mechanical properties within prepared fibrous porous materials, meaning they cannot then meet various criteria for practical use. Therefore, improving the dispersibility of fibers in the slurry is crucial to preparing the desirable alumina–silica fibrous porous materials with uniform structure and excellent performance.

**Figure 3:**
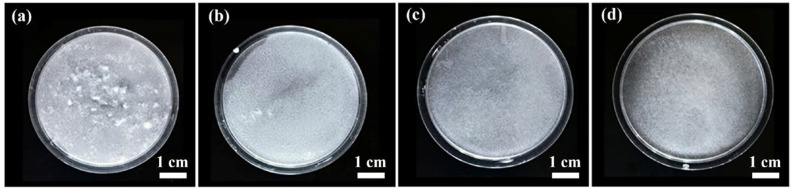
Digital photographs of the dispersion of ASF2 in ASF2 slurry without (**a**) and with (**b**) the addition of SHMP dispersant (0.4 wt%). Digital photographs of dispersion of ASF1 (**c**) and ASF3 (**d**) in the corresponding slurry with the addition of SHMP dispersant (0.4 wt%).

**Figure 4:**
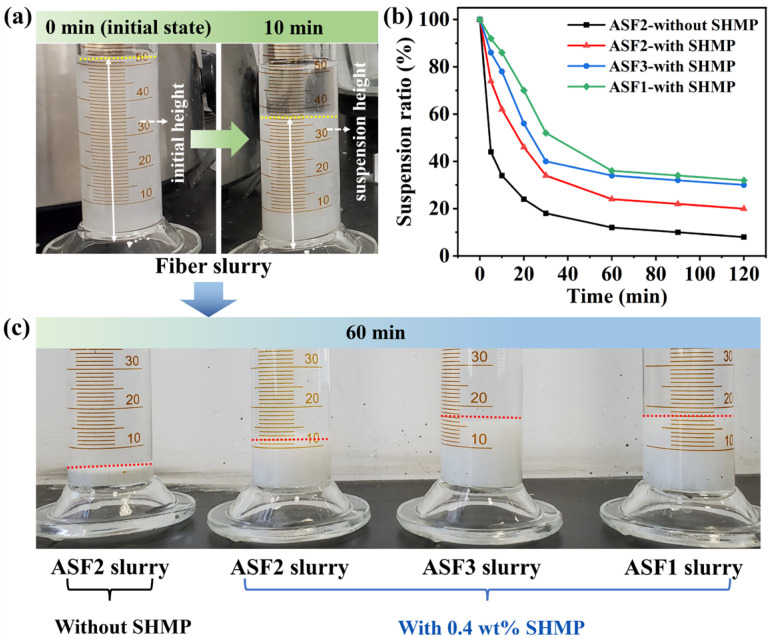
(**a**) Digital photographs of fiber slurry after standing for 0 and 10 min. (**b**) The suspension ratios for sedimentation time ranging from 0 to 120 min of ASF2 slurries without SHMP addition, and ASF1, ASF2, and ASF3 slurries with SHMP addition. (**c**) Digital photographs of the ASF2 slurry without SHMP addition, and ASF1, ASF2, and ASF3 slurries with SHMP addition after standing for 60 min. (The solid white arrows refers to the height of fiber settlement, and the colored dotted lines indicate the liquid level of fiber settlement).

The effect of SHMP dispersant on the dispersibility of fibers was studied, and the dispersibility of ASF1, ASF2, and ASF3 in corresponding slurry samples was studied, as shown in Figure 3a–d and Figure 4a–c. Figure 3a–d was captured after the addition of dispersant into the slurry and stirring for 30 min, followed by allowing it to settle undisturbed for 10 min.

In the ASF2 slurry without the addition of SHMP dispersant (0.4 wt%), a significant fiber agglomeration was observed, as shown in Figure 3a. The existence and maldistribution of fiber flocs formed by agglomeration indicate the poor dispersion of ASF2 in the slurry. After introducing SHMP dispersant (0.4 wt%) into the ASF2 slurry, Figure 3b shows that although a small amount of fiber aggregation could still be observed in the ASF2 slurry, the dispersibility of ASF2 was significantly improved. With the addition of SHMP dispersant, the ASF1 slurry and ASF3 slurry did not feature obvious fiber flocs when compared with the ASF2 slurry, as shown in Figure 3c,d, and the fiber agglomeration almost completely disappeared. The distributions of ASF1 and ASF3 were uniform, indicating that both ASF1 and ASF3 had excellent dispersibility in the corresponding slurry.

The dispersion of three types of alumina–silica fibers in the slurries was further characterized by sedimentation experiments, and the suspension ratio of fibers in the slurry was calculated. As shown in Figure 4a, the suspension ratio was the ratio of the suspension height to the initial height of the fiber slurry. The driving force of sedimentation is the gravitational or inertial centrifugal force exerted on suspended particles, which is positively associated with the particle size. Smaller particle sizes will contribute to a decrease in sedimentation rate and an increase in suspension ratio and suspension height. As shown in Figure 4b,c, the suspension ratios of three kinds of fibers exhibit a decreasing trend as the sedimentation time increases. Once the sedimentation time exceeds 60 min, the suspension ratios remain stable with only a slight decrease.

In the preparation process, after the slurry is poured into the cylindrical metal mold, the fibers in the slurry will settle freely, and gradually overlap to form a stable structure with time increasing. Based on the results of sedimentation experiments, the settling height of the fibers reaches a state of stability after standing for 1 h. Further extension of the sedimentation time shows minimal changes in suspension height. Considering both the preparation process and structural stability, 1 h is selected as the optimal sedimentation time.

For ASF2 slurry without SHMP dispersant addition, the suspension ratio decreases to 44% and 18% at the sedimentation time of 5 min and 30 min, respectively. After adding 0.4 wt% SHMP dispersant to ASF2 slurry, the suspension ratio and suspension height increase, and the sedimentation rate significantly decreases. When the sedimentation time reaches 60 min, the suspension ratios and suspension heights of ASF1 and ASF3 are significantly higher than ASF2. Results indicate solids in both ASF1 and ASF3 slurries have smaller sizes than those in ASF2 slurries, corresponding to reduced fiber agglomeration. Thus, the dispersibility of ASF1 and ASF3 is superior to that of ASF2. The type and concentration of dispersants utilized in ASF2 slurry remain consistent with those employed in ASF1 and ASF3 slurries. The used amount of the dispersant is appropriate for ASF1 and ASF3, but not for ASF2. Other types and concentrations of dispersants may be effective for ASF2, and we will further investigate them in future work.

The bulk density and thermal conductivity of three kinds of alumina–silica fibrous porous materials (ASFPM1, ASFPM2, and ASFPM3) are displayed in Table 2. ASFPM1 showed the lowest bulk density of 0.11 g·cm^−3^, the lowest fiber volume fraction of 3.4%, and the lowest thermal conductivity of 0.0486 W·m^−1^·K^−1^, which indicates the good dispersion of ASF1 in the slurry. ASFPM2 exhibited the highest bulk density of 0.17 g·cm^−3^, the highest fiber volume fraction of 5.3%, and the highest thermal conductivity of 0.0721 W·m^−1^·K^−1^, and these results may be attributed to the uneven structure formed by the poor dispersion of ASF2 in the slurry. Such results indicate that all three alumina–silica fibrous porous materials have low bulk density and excellent thermal insulation performance.

### 3.2. Evolution of the Composition, Structure, and High-Temperature Resistance and Thermal Stability of ASFPM1, ASFPM2, and ASFPM3

Samples of ASFPM1, ASFPM2, and ASFPM3 were subjected to heat treatment at different temperatures (1100 °C, 1300 °C, and 1500 °C) for 2 h. Subsequently, the phase composition and microstructure of the products were analyzed to evaluate their high-temperature resistance and thermal stability.

**Figure 5:**
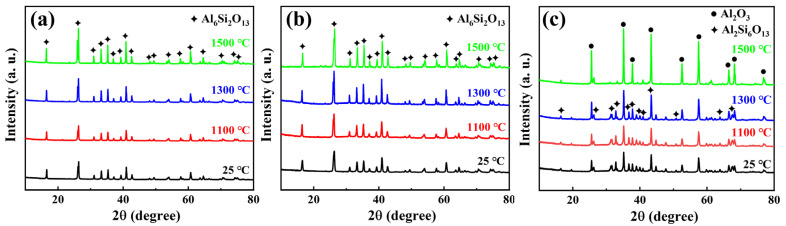
XRD patterns of (**a**) ASFPM1, (**b**) ASFPM2, and (**c**) ASFPM3 before (25 °C) and after heat treatment at different temperatures (1100 °C, 1300 °C, and 1500 °C).

Figure 5a shows X-ray diffraction patterns of ASFPM1 before and after heat treatment at different temperatures. According to our XRD results, the phase composition of ASFPM1 at room temperature (25 °C) is Al_6_Si_2_O_13_ (mullite, JCPDS No. 79-1275). After heat treatment, there was no significant change in the phase of the ASFPM1; however, we observed that the shape of the diffraction peaks became slightly sharper, indicating the excellent high-temperature resistance and thermal stability of the ASFPM1.

The X-ray diffraction patterns of ASFPM2 before and after heat treatment at different temperatures are presented in Figure 5b. Similar to ASFPM1, the phase compositions of ASFPM2 before and after heat treatment mainly consist of the Al_6_Si_2_O_13_ (mullite, JCPDS No. 79-1275) phase. However, compared to ASFPM1, the characteristic peak of Al_6_Si_2_O_13_ is more intense. The mass fractions of Al_2_O_3_ and mullite in ASF3 after heat treatment at 1500 °C were calculated using XRD data. The mass fraction of Al_2_O_3_ and mullite is 92.5% and 7.5%, respectively.

Figure 5c exhibits the X-ray diffraction patterns of ASFPM3 before and after heat treatment at different temperatures. The XRD results demonstrated that the phase composition of ASFPM3 at room temperature comprised Al_2_O_3_ (JCPDS No. 71-1683) and Al_6_Si_2_O_13_ (mullite, JCPDS No. 79-1458). After heat treatment at 1100 °C or 1300 °C, the diffraction patterns of ASFPM3 were relatively similar to the diffraction patterns of ASFPM3 at 25 °C. When the temperature of the heat treatment was 1500 °C, the shape of the diffraction peaks became sharp, indicating an increase in the crystallinity of the phases. In addition, no obvious change in the phases proves that ASFPM3 possesses excellent thermal stability and high-temperature resistance.

**Figure 6:**
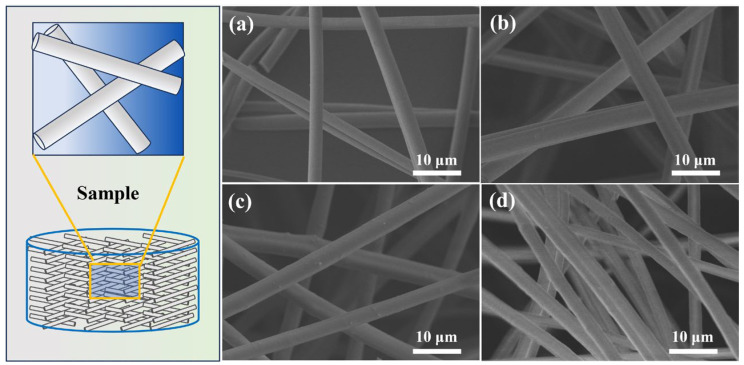
Microstructures of ASFPM1 (**a**), ASFPM3 (**b**) and ASFPM2 (**c**,**d**) at room temperature (25 °C).

The microstructures of different aluminum silicon fibrous porous materials (ASFPM1, ASFPM2, and ASFPM3) are shown in Figure 6a–d. All samples exhibited a three-dimensional fibrous porous structure. Overall, the orientation and arrangement of alumina–silica fibers vary, and fibers appear to overlap, being randomly distributed. In addition, adjacent fibers overlap relatively loosely, forming numerous large pores. ASFPM1 and ASFPM3 exhibit uniform three-dimensional skeletons benefiting from the uniform distribution of fibers at different positions in the samples. However, in ASFPM2, the distribution and density of fibers in different positions are different, indicating an uneven fibrous network structure. This outcome can mainly be attributed to the poor dispersibility of ASF2 in the slurry, as the type and concentration of used dispersants are not appropriate for ASF2.

**Figure 7:**
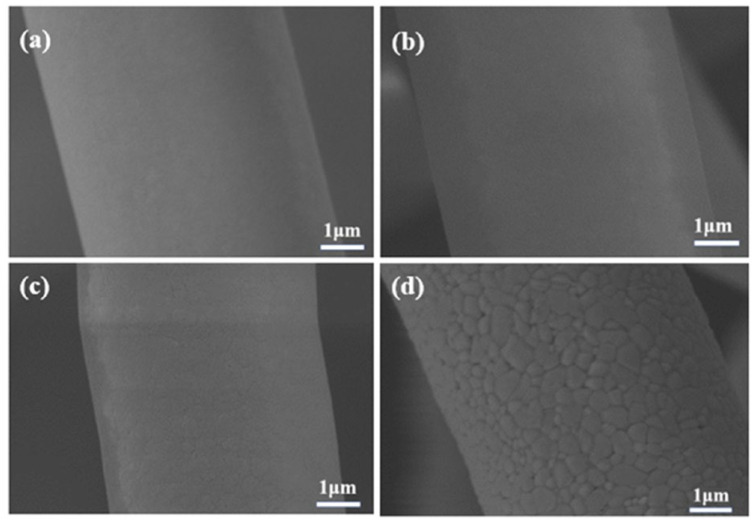
SEM images of ASF1 before (**a**) and after heat treatment at (**b**) 1100 °C, (**c**) 1300 °C, and (**d**) 1500 °C.

Microstructures of ASF1 before (25 °C) and after heat treatment at different temperatures (1100 °C, 1300 °C, and 1500 °C) are displayed in Figure 7a–d. As shown in Figure 7a,b, the surface of ASF1 is smooth and remains stable at both 25 °C and 1100 °C. After heat treatment at 1300 °C, the local surface of fibers exhibits the initial signs of grain growth. (Figure 7c). When the temperature of heat treatment rises to 1500 °C, the phenomenon of grain growth on the fiber surface is more significant. The relatively smooth fiber surface becomes rougher due to grain growth, but the fibers maintain a stable structure, as shown in Figure 7d.

**Figure 8:**
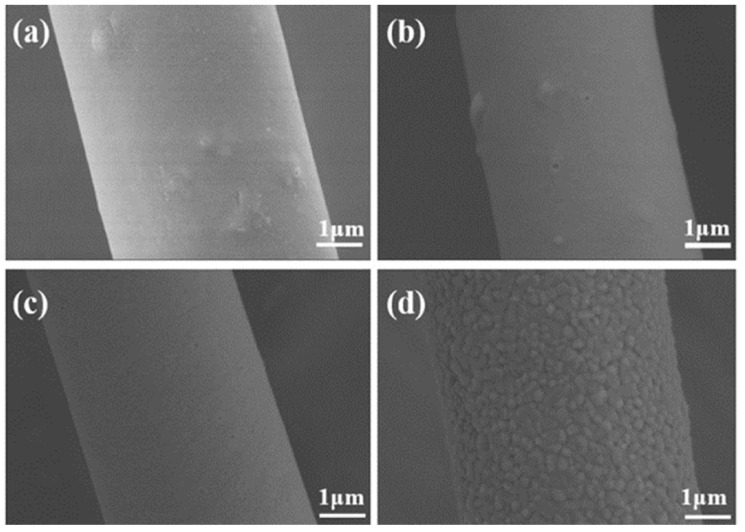
SEM images of ASF2 before (**a**) and after heat treatment at (**b**) 1100 °C, (**c**) 1300 °C, and (**d**) 1500 °C.

The microstructures of ASF2 before (25 °C) and after heat treatment at different temperatures (1100 °C, 1300 °C, and 1500 °C) are displayed in Figure 8a–d. A small number of protruding structures can be observed on the relatively smooth fiber surface in Figure 8a,b. After heat treatment at 1300 °C, the protruding structures gradually disappear, and the grains on the surface of the fibers begin to grow (Figure 8c); this event may be related to changes in the phase of ASF2. After heat treatment at 1500 °C, the degree of particle growth further increases. Plenty of nanosized grains cover the whole fiber surface, and the fiber surface becomes rough, as shown in Figure 8d.

**Figure 9:**
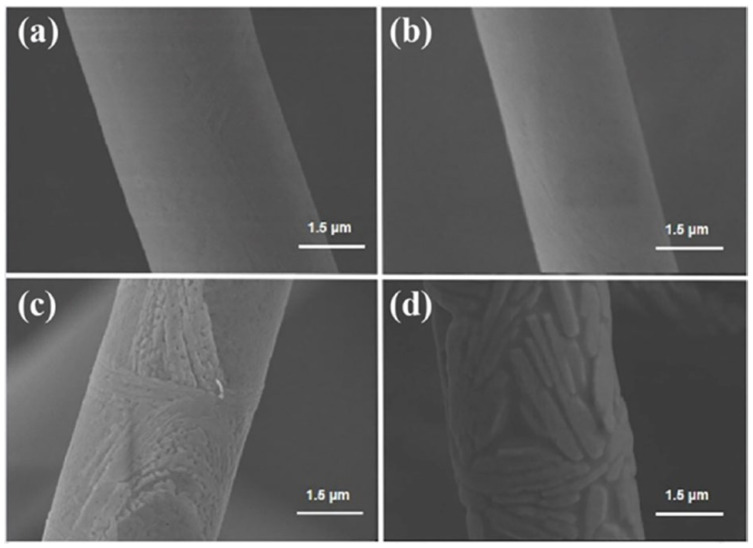
SEM images of ASF3 before (**a**) and after heat treatment at (**b**) 1100 °C, (**c**) 1300 °C, and (**d**) 1500 °C.

Figure 9a–d shows the microstructures of ASF3 before (25 °C) and after heat treatment at different temperatures (1100 °C, 1300 °C, and 1500 °C). As in the case of ASF1 and ASF2, the fiber surface is smooth, and the structure remains unchanged at 25 °C and 1100 °C. As the heat treatment temperature rises to 1300 °C (Figure 9c), the particle growth of fibers occurs, producing a rough surface. When the heat treatment temperature rises to 1500 °C, the particle growth of fibers becomes more pronounced, as shown in Figure 9d. Unlike the irregular and granular grains in ASF1 and ASF2, the grains that accumulate in ASF3 are larger, and the gathered grains form a long strip-like shape. According to the XRD results, mullite is identified as the crystallization phase of ASF1 and ASF2, while corundum is identified as the crystallization phase of ASF3. The variation in grain morphology might be attributed to the different phase compositions and silica content of the ASF1, ASF2, and ASF3.

To summarize, the phase composition of all the samples remains unchanged below 1300 °C, and the structure of all the samples remains stable even during heat treatments at up to 1500 °C. In terms of phase composition and microstructure, our results indicate that all three products have excellent thermal stability and high-temperature resistance.

### 3.3. Thermal Insulation Performance and Compressive Rebound Capacity

Using a constant testing temperature, the thermal insulation performance of different samples can be evaluated and compared through analysis of the backside temperature of samples.

**Figure 10:**
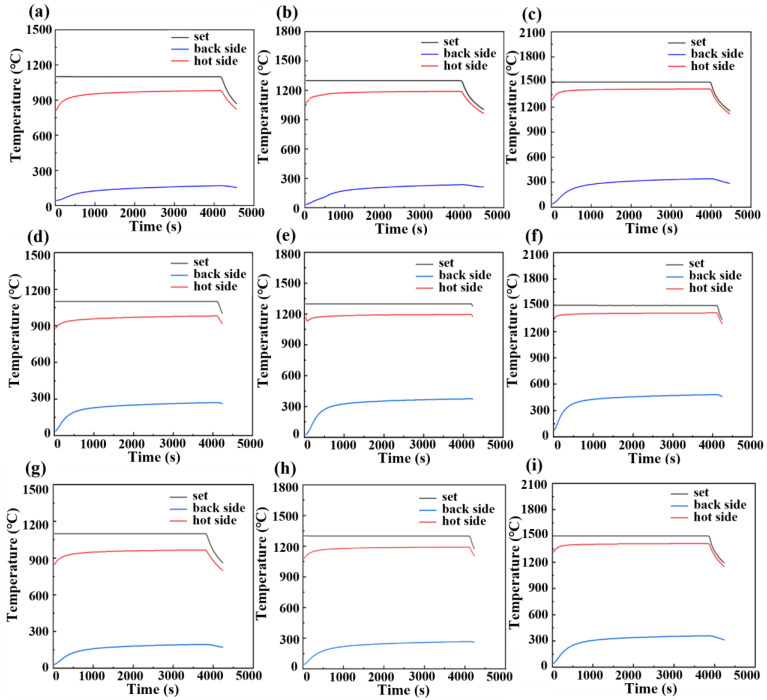
Real-time temperature–time curves of (**a**–**c**) ASFPM1, (**d**–**f**) ASFPM2, and (**g**–**i**) ASFPM3 during backside temperature testing at different test temperatures of 1100 °C, 1300 °C, and 1500 °C.

Figure 10a–c shows real-time temperature–time curves of ASFPM1 during backside temperature testing at different test temperatures. When the test temperature is 1100 °C, 1300 °C, and 1500 °C, the highest backside temperature is 170.5 °C, 237.9 °C, and 342.5 °C, respectively. After the unilateral heating program ends, the temperature of the hot side surface begins to decrease. Because it is influenced by the speed at which the temperature transfers and its distribution, the backside temperature of the sample still slightly increases. Approximately 1 min after the unilateral heating program ends, the backside temperature reaches its peak and then begins to decrease. The relatively low backside temperature that we recorded indicates that ASFPM1 has excellent thermal insulation capacity, even in high-temperature environments of up to 1500 °C.

Figure 10d–f shows the real-time temperature–time curves of ASFPM2 during backside temperature testing at different test temperatures. When the test temperature is 1100 °C, 1300 °C, and 1500 °C, the highest backside temperature is 272.4 °C, 376.0 °C, and 482.5 °C, respectively. All the backside temperatures of ASFPM2 are more than 100 °C higher than those of ASFPM1 when the test temperature is 1100 °C, 1300 °C, and 1500 °C. This result indicates that the thermal insulation performance of ASFPM1 is superior to that of ASFPM2.

Figure 10g–i shows real-time temperature–time curves of ASFPM3 during backside temperature testing at different test temperatures. When the test temperature is 1100 °C, 1300 °C, and 1500 °C, the highest backside temperature is 196.5 °C, 268.0 °C, and 361.6 °C, respectively. As indicated in Table 3, when the test temperature is 1100 °C, 1300 °C, and 1500 °C, all the backside temperatures of ASFPM3 are around 20 °C higher than those of ASFPM1. Meanwhile, all the backside temperatures of ASFPM3 are more than 70 °C lower than those of ASFPM2, which indicates that the thermal insulation performance of ASFPM3 is better than that of ASFPM2. Among the three products, ASFPM1 therefore demonstrates the best thermal insulation performance.

**Figure 11:**
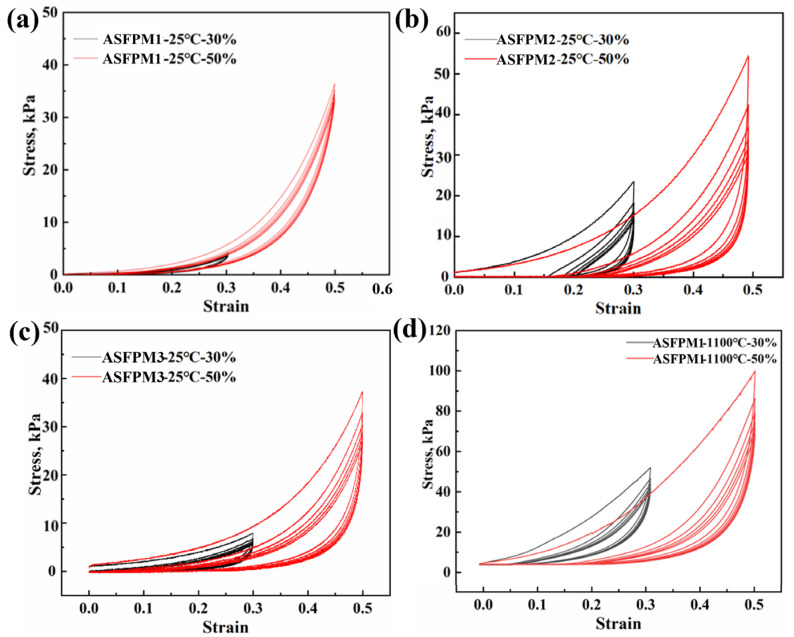
Compressive rebound curves at 30% and 50% strain of (**a**) ASFPM1, (**b**) ASFPM2, and (**c**) ASFPM3 at 25 °C. (**d**) Compressive rebound curves at 30% and 50% strain of ASFPM1 after heat treatment at 1100 °C.

In addition to thermal insulation, the capacity for a compressive rebound is a key indicator of the fibrous porous material’s efficacy. Figure 11a–d shows the compressive rebound stress–strain curves of the products (ASFPM1, ASFPM2, and ASFPM3). When compressed under 30% and 50% strain at room temperature (25 °C), the rebound rates of ASFPM1, ASFPM2, and ASFPM3 are 92.9% and 87.5%, 52.6% and 45.2%, and 64.7% and 48.3%, respectively. After compression, all the samples show incomplete rebound. The rebound rate of ASFPM1 is the highest, and the rebound rate of ASFPM2 is the lowest, indicating that ASFPM1 has excellent capacity for compressive rebounds. Heat treatment at high temperatures not only affects the phase composition and microstructure of alumina–silica fibrous porous materials; it also affects their compressive rebound characteristics. Thus, the capacity of ASFPM1 for compressive rebound after heat treatment at 1100 °C was investigated, as shown in Figure 11d. When compressed under 30% and 50% strain at 1100 °C, the rebound rate of ASFPM1 is 84.5% and 80.2%, respectively. After ASFPM1 is heated at 1100 °C, the decrease in the rebound rate might be due to the oxidative volatilization of organics or the higher crystallization of a few fibers after heat treatment at 1100 °C.

**Figure 12:**
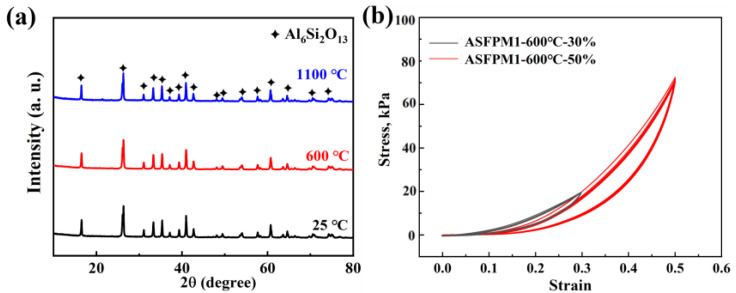
(**a**) XRD patterns of ASFPM1 before (25 °C) and after heat treatment at different temperatures (600 °C and 1100 °C). (**b**) Compressive rebound curves at 30% and 50% strain of ASFPM1 after heat treatment at 600 °C.

The phase composition analysis and compression rebound tests of ASFPM1 were conducted after heat treatment at 600 °C to further investigate the impact of organics volatilization and fiber crystallization on the loss in the rebound. As shown in Figure 12a, the XRD patterns of ASFPM1 before and after heat treatment at 600 °C are essentially the same, with a single mullite phase as the main phase composition. It indicates that there is no phase change in ASFPM1 after heat treatment at 600 °C while the organics are removed. Figure 12b displays the compression rebound curves of ASFPM1 after heat treatment at 600 °C. When compressed under 30% and 50% strain at 600 °C, the rebound rate of ASFPM1 is 87.6% and 83.1%, respectively. Compared to the ASFPM1 without heat treatment, the rebound rate decreases but still remains higher than the rebound rate of ASFPM1 after heat treatment at 1100 °C. Combined with the SEM results, it demonstrates that either the organic volatilization or the higher crystallization of the fibers can lead to the decline in the rebound rates of the products.

Compared to the ASFPM1 without heat treatment, the structure of ASFPM1 after heat treatment becomes more brittle and prone to breaking when pressure is applied. As the number of cycles increases, the degree of fracturing in the samples continues to increase, leading to a transition from a lap structure composed of high-aspect-ratio fibers to a stacked structure composed of low-aspect-ratio fibers. Therefore, the number of overlapping junctions between fibers, the density of the sample, and the peak stress increase, while the rebound rate and the elasticity decrease.

The thickness, peak stress, bulk density, and fiber volume fraction of ASFPM1 before and after heat treatments at 600 °C and 1100 °C are shown in Table S1. The peak stress (50% strain) increases with the increase in the heat treatment temperature. After heat treatment at 600 °C, ASFPM1 exhibits a slight expansion in volume, an increase in thickness, and a decrease in bulk density and fiber volume fraction, which is mainly attributed to the organics volatilization. When the heat treatment temperature is 1100 °C, ASFPM1 exhibits a significant volumetric shrinkage and reduction in thickness. Compared to ASFPM1 without heat treatment, the bulk density and fiber volume fraction increase.

## 4. Conclusions

In summary, mullite fibrous porous materials were fabricated via molding methods combined with vacuum filtration using mullite fibers. We investigated the high-temperature resistance and thermal stability of samples composed of three fibers with different proportions of alumina. The phase composition and particle growth of fibers showed no obvious change while the temperature of heat treatment remained below 1300 °C. After heat treatment temperatures reached 1500 °C, all the samples still retained a stable fibrous structure. The compressive rebound rate of the products at 25 °C was up to 92.9% and decreased only by 8.4 percentage points after the samples were heated to 1100 °C. In addition, the backside temperature of the as-prepared samples was as low as 361.6 °C at 1500 °C. The as-prepared mullite fibrous porous materials exhibited good integration with excellent high-temperature resistance, thermal stability, thermal insulation capacity, and compressive rebound characteristics, thus indicating that they may play an important role as mullite fiber felt materials in advanced flexible external thermal insulation blankets.

## Figures and Tables

**Table 1:** Component, fiber diameter, and phase composition of the three kinds of alumina–silica fibers.

Sample	Mass Fraction of Al_2_O_3_ (%)	Mass Fraction of SiO_2_ (%)	Fiber Diameter (μm)	Phase Composition
ASF1	81.7	17.8	3.0–5.0	Mullite
ASF2	83.6	15.2	3.0–5.0	Mullite
ASF3	96.9	2.58	3.0–5.0	Mullite, Al_2_O_3_

**Table 2:** Bulk density, fiber volume fraction, and thermal conductivity of ASFPM1, ASFPM2, and ASFPM3.

Sample	Bulk Density (g·cm^−3^)	Fiber Volume Fraction (%)	Thermal Conductivity (W·m^−1^·K^−1^)
ASFPM1	0.11	3.4	0.0486
ASFPM2	0.17	5.3	0.0721
ASFPM3	0.13	4.1	0.0511

**Table 3:** Backside temperatures of products (ASFPM1, ASFPM2, and ASFPM3) at different test temperatures (1100 °C, 1300 °C, and 1500 °C).

Sample	1100 °C	1300 °C	1500 °C
ASFPM1	170.5 °C	237.9 °C	342.5 °C
ASFPM2	272.4 °C	376.0 °C	482.5 °C
ASFPM3	196.5 °C	268.0 °C	361.6 °C

## Data Availability

The raw data supporting the conclusions of this article will be made available by the authors on request.

## Supplementary Materials

The following supporting information can be downloaded at: https://www.mdpi.com/article/10.3390/ma17133235/s1, Figure S1: EDS mapping of ASF2 after heat treatment at 1500 °C; Table S1: Thickness, peak stress, bulk density and the fiber volume fraction of ASFPM1 before and after heat treatment at different temperatures.
