# Supplementary material for: Preparation and Properties of Elastic Mullite Fibrous Porous Materials with Excellent High-Temperature Resistance and Thermal Stability
Source: Materials (Basel). 2024 Jul 1;17(13):3235. doi: 10.3390/ma17133235 (PMC11242305; doi:10.3390/ma17133235)
Supplement: Supplementary file 1 [file materials-17-03235-s001.zip › materials-2954287-supplementary.pdf]

materials

Supporting Information

Preparation and Properties of Elastic Mullite Fibrous Porous Materials with Excellent High-Temperature Resistance and Thermal Stability

Xiang Zhang [1,2], Xueying Zhang [1,*], Zhongyan Wang [1], Yunjia Xue [3], Anran Guo [1], Liwen Yan [1], Feng Hou [1] and Jiachen Liu [1,*]

[1] Key Lab of Advanced Ceramics and Machining Technology of Ministry of Education, School of Materials Science and Engineering, Tianjin University, Tianjin 300072, China; zx_koj@163.com (X.Z.); 18131126553@163.com (Z.W.); arguo@tju.edu.cn (A.G.); lwyan@tju.edu.cn (L.Y.); houf@tju.edu.cn (F.H.)
[2] Beijing Institute of Astronautical System Engineering, Beijing 100076, China
[3] Beijing Hangxing Machinery Co., Ltd., Beijing 100013, China; xueyj@tju.edu.cn
* Correspondence: zxy_92@163.com (X.Z.); jcliutju@tju.edu.cn (J.L.)

Table S1. Thickness, peak stress, bulk density and the fiber volume fraction of ASFPM1 before and after heat treatment at different temperatures.

Sample	Thickness (mm)	Peak stress (kPa, 50% strain)	Bulk density ($g \cdot cm^{-3}$)	Fiber volume fraction (%)
ASFPM1-25 °C	31.5	32.9	0.11	3.4
ASFPM1-600 °C	34.6	72.5	0.10	3.1
ASFPM1-1100 °C	28.9	101.4	0.12	3.8

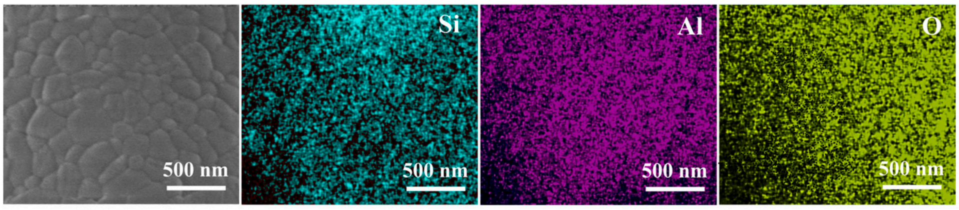

Figure S1. EDS mapping of ASF2 after heat treatment at 1500 °C.

The EDS mapping of the fiber surface in ASF2 is shown in Figure S1. Results show that ASF2 after heat treatment at 1500°C includes Si, Al, and O elements, and all the elements are distributed evenly on the surface of the fiber surface. Combined with the XRD results, the distribution of elements corresponds to the mullite phase.
